# Pleuroperitoneal Communication following Right Hepatectomy: Successful Treatment with a Combined Thoracoscopic and Laparoscopic Approach

**DOI:** 10.70352/scrj.cr.25-0280

**Published:** 2025-09-06

**Authors:** Sota Nakamura, Shohei Yoshiya, Kensaku Ito, Noriaki Yamamoto, Ippei Kawada, Kazuhiro Tada, Yosuke Kuroda, Fumitaka Yoshizumi, Hidenori Kouso, Kentaro Iwaki, Shoji Hiroshige, Hideya Takeuchi, Yo-Ichi Yamashita, Kengo Fukuzawa, Tomoharu Yoshizumi

**Affiliations:** 1 Department of Surgery and Science, Graduate School of Medical Sciences, Kyushu University, Fukuoka, Fukuoka, Japan; 2 Department of Surgery, Oita Red Cross Hospital, Oita, Oita, Japan; 3 Department of Pathology, Oita Red Cross Hospital, Oita, Oita, Japan

**Keywords:** pleuroperitoneal communication, diaphragmatic hernia, malignant ascites, thoracic and laparoscopic surgery

## Abstract

**INTRODUCTION:**

Pleuroperitoneal communication (PPC) is an abnormal connection between the thoracic and abdominal cavities, allowing ascitic fluid to migrate into the pleural space and cause pleural effusion. Although PPC is a recognized complication in peritoneal dialysis patients, it is rarely reported as a postoperative complication.

**CASE PRESENTATION:**

A 54-year-old female patient with a history of right hemicolectomy and right hepatectomy for ascending colon cancer and metastatic liver tumor developed persistent right pleural effusion 3 months postoperatively. She also had ovarian metastases, peritoneal dissemination, and malignant ascites. Despite treatment with diuretics and 2 attempts at pleurodesis, the pleural effusion persisted. A combined thoracoscopic and laparoscopic approach was performed. Intraoperatively, a diaphragmatic hernia with a pinhole defect was identified, suggesting migration of ascitic fluid into the thoracic cavity. Resection of the hernia sac and suture closure of the diaphragmatic defect were carried out. Postoperatively, the pleural effusion resolved, and her dyspnea improved.

**CONCLUSIONS:**

In case of refractory pleural effusion following hepatectomy, PPC due to diaphragmatic defects should be considered, particularly in the presence of ascites. A combined thoracoscopic and laparoscopic approach is a viable surgical option for definitive treatment.

## Abbreviations

CO_2_
: carbon dioxide gas
PPC
: pleuroperitoneal communication

## INTRODUCTION

Pleuroperitoneal communication (PPC) is an abnormal connection between the thoracic and abdominal cavities.^1)^ Persistent migration of ascitic fluid into the thoracic cavity through PPC, driven by negative intrapleural pressure, can result in pleural effusion and increase the risk of respiratory failure.^2)^ PPC is a rare condition, observed in approximately 2% of patients undergoing peritoneal dialysis, but it is seldom recognized as a postoperative complication.^1)^ Diaphragmatic hernia is a well-known complication following right hepatectomy, occurring in approximately 0.75% of cases.^3)^ However, to our knowledge, there have been no previous reports of PPC occurring after right hepatectomy.

Here, we present a unique case of PPC following right hepatectomy, presumed to result from a coexistence of diaphragmatic hernia and malignant ascites, which was successfully treated with thoracoscopic and laparoscopic diaphragmatic repair.

## CASE PRESENTATION

A 54-year-old female patient presented with exertional dyspnea. She had undergone right hemicolectomy for ascending colon cancer with bilobar liver metastases 8 months earlier, followed by Associating Liver Partition and Portal vein embolization for Staged hepatectomy 6 months before this presentation. Three months postoperatively, she developed ovarian metastases (Krukenberg tumor), peritoneal dissemination, and malignant ascites, necessitating hospitalization for ascitic drainage. Although she initially declined chemotherapy at recurrence, she later opted for a mild regimen and was treated with tegafur/gimeracil/oteracil plus bevacizumab.

**Fig. 1:**
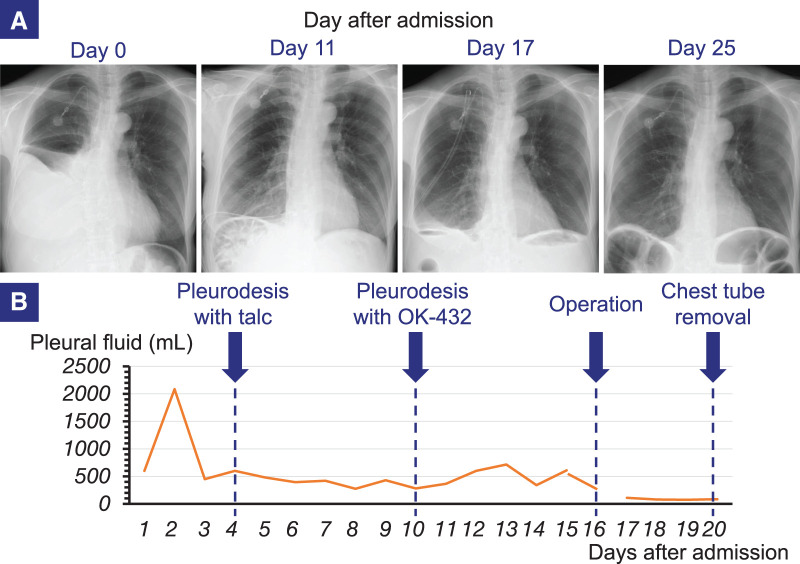
Preoperative changes in chest radiography and pleural drainage volume. (**A**) Chest radiograph on admission demonstrating right pleural effusion. Post-drainage radiograph showing adequate right lung expansion. Postoperative chest radiograph showing adequate right lung re-expansion. No recurrence of pleural effusion was observed following chest tube removal. (**B**) The perioperative drainage volume from the chest tube is shown in the graph. Despite 2 attempts at pleurodesis, there was no significant reduction in pleural effusion. Postoperatively, the pleural effusion markedly decreased, and the chest tube was removed on POD 3.

**Fig. 2:**
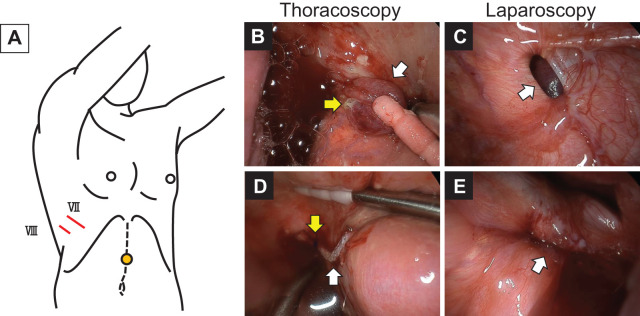
Surgical setup and intraoperative thoracoscopic and laparoscopic findings. (**A**) The chest tube placed at the 8th intercostal space along the midaxillary line was removed, and the incision was extended to serve as the initial thoracoscopic camera port. Under thoracoscopic guidance, a 4-cm utility incision was made along the anterior axillary line at the 7th intercostal space, allowing a 2-port approach. Given the history of prior laparotomy and anticipated extensive intra-abdominal adhesions, preoperative abdominal ultrasound was performed to assess adhesion sites. A camera port was placed at a site where mild ascites provided sufficient separation between the abdominal wall and the intra-abdominal organs (yellow circle). (**B**) Thoracoscopy revealed a 1-cm diaphragmatic hernia sac (white arrow). A pneumoperitoneum test using CO_2_ insufflation demonstrated air leakage from a pinhole defect within the hernia sac (yellow arrow), confirming the presence of a pleuroperitoneal communication. (**C**) Laparoscopy identified a 1-cm diaphragmatic defect in the central tendinous portion of the right diaphragm, without evidence of organ herniation (white arrow). (**D**) The hernia sac was resected using an endoscopic stapler (white arrow). Persistent air leakage on repeated pneumoperitoneum testing necessitated additional sutured closure (yellow arrow). (**E**) Laparoscopic assessment confirmed the absence of herniated abdominal organs (white arrow).

On physical examination, mild abdominal distention was noted. Laboratory findings revealed a white blood cell count of 6.6 × 10^6^/L, an albumin level of 39 g/L, and a C-reactive protein level of 0.7 mg/L. Chest radiography showed right pleural effusion (**Fig. 1A**), prompting chest tube placement. The lung expanded well following drainage (**Fig. 1A**). Cytological analysis of the pleural fluid revealed an exudative effusion with markedly elevated tumor markers, suggesting malignant pleuritis. Despite 2 attempts at pleurodesis with talc and OK-432, the pleural effusion persisted (**Fig. 1B**). During treatment, the patient’s abdominal distension improved, revealing a palpable Krukenberg tumor. These findings suggested that the pleural effusion resulted from the migration of malignant ascites through a PPC. Consequently, we performed surgical treatment. Under general anesthesia, single-lung ventilation was achieved using a double-lumen endotracheal tube, with the patient placed in a left-sided semi-lateral decubitus position (**Fig. 2A**). A 2-cm skin incision was made along the mid-axillary line at the 8th intercostal space, and a 4-cm incision was made along the anterior axillary line at the 7th intercostal space, allowing a 2-port thoracoscopic approach (**Fig. 2A**). Thoracoscopy revealed a 1.5-cm hernia sac protruding through the right diaphragm (**Fig. 2B**). No significant pleural nodules suggesting dissemination were observed. The preoperative plan was to initially observe the diaphragm thoracoscopically and proceed with repair if the PPC site could be clearly identified; if not, a laparoscopic approach would be added. However, the diaphragmatic hernia alone was not sufficient to confirm it as the definitive site of the PPC, and the possibility of organ herniation also could not be ruled out; therefore, an additional laparoscopic approach was required. A 2-cm vertical incision was made over the previous upper midline abdominal scar, and a camera port was inserted for carbon dioxide gas (CO_2_) insufflation (**Fig. 2A**). Upon insufflation, bubbles were observed leaking from a pinhole defect in the hernia sac into the thoracic cavity (**Fig. 2B**), identifying the PPC site. Laparoscopy revealed a diaphragmatic defect through which the hernia sac protruded into the thoracic cavity (**Fig. 2C**). No bowel herniation was observed. After confirming the absence of herniated organs on laparoscopy, the hernia sac was resected using an endoscopic stapler from the thoracic side (**Fig. 2D**, **2E**). A leak test with normal saline in the thoracic cavity and CO_2_ insufflation in the abdominal cavity revealed persistent air leakage. The air leak point was closed with interrupted 2-0 non-absorbable monofilament sutures (**Fig. 2D**). A repeat leak test confirmed no air leakage. Pathological examination of the resected hernia sac revealed pleural tissue with infiltration of inflammatory cells including neutrophils, hemorrhage, and deposition of cholesterol crystals. No malignancy or fibrosis was observed.

The patient’s postoperative course was uneventful. The chest tube was removed on POD 3, with no recurrence of pleural effusion (**Fig. 1A**). However, ascitic fluid, which could no longer drain into the thoracic cavity, accumulated, leading to abdominal distension. This was managed with diuretics, hydromorphone hydrochloride, and cell-free and concentrated ascites reinfusion therapy. The patient was transferred to a hospice care on POD 12 for best supportive care.

## DISCUSSION

PPC arises from an abnormal connection between the thoracic and abdominal cavities. While it can result from a visibly large diaphragmatic defect, it may also occur through an apparently intact diaphragm.^1)^ In 1998, Dr. Kirschner described the porous diaphragm syndrome, identifying small pinholes, slits, or webs in the central tendinous portion of the diaphragm as potential sites of PPC.^4)^ These defects may be congenital or acquired due to factors such as an increased intra-abdominal pressure or diaphragmatic tissue disruption.^1)^ PPC predominantly affects in the right diaphragm.^5)^

PPC becomes a clinical concern when ascitic fluid accumulates in the thoracic cavity, leading to symptoms such as dyspnea.^2)^ The most common forms of PPC include peritoneal dialysis-associated hydrothorax and hepatic hydrothorax, both of which are considered as a part of the porous diaphragm syndrome.^5,6)^ PPC in peritoneal dialysis patients has been well documented, with the underlying defects typically presenting as small slits, diaphragmatic blebs, or small pores.^7–11)^

To better understand the clinical spectrum of PPC, we conducted a literature review using the keyword “pleuroperitoneal communication” in the PubMed database, covering the period from 1980 to June 2025. A total of 131 cases with sufficient clinical information were identified.^2,7–66)^

**Fig. 3:**
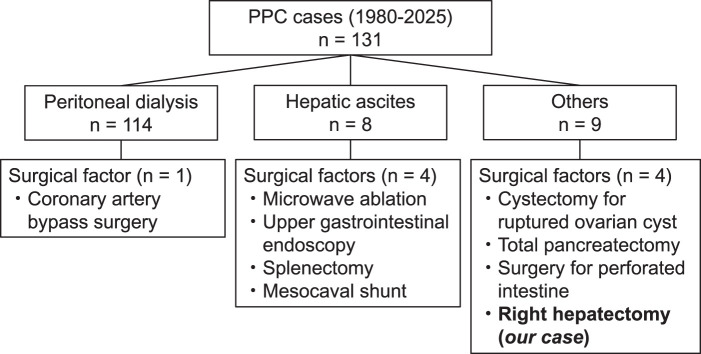
Etiological distribution of reported cases of pleuroperitoneal communication (PPC) between 1980 and 2025. Most cases were associated with peritoneal dialysis (n = 114) or hepatic hydrothorax (n = 8). PPC following right hepatectomy was reported only in the present case.

**Fig. 4:**
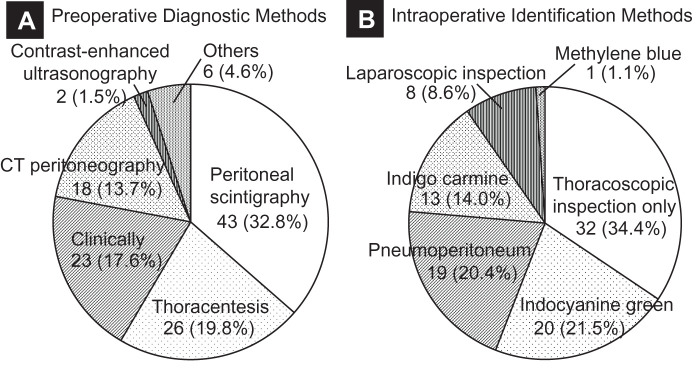
Diagnostic and intraoperative methods for identifying pleuroperitoneal communication (PPC). (**A**) Preoperative diagnostic methods for PPC in 131 reported cases. Among these, diagnostic methods were not described in 13 cases, leaving 118 cases for which the methods were available and included in the pie chart. In peritoneal dialysis-related cases, imaging studies such as peritoneal scintigraphy and CT peritoneography were commonly used. A comparable number of cases, including the present case, were diagnosed based on clinical symptoms or pleural effusion findings. (**B**) Intraoperative methods for identifying the diaphragmatic defect. Of the 131 cases, 102 underwent surgery, with detection methods described in 93. Dye-based techniques using indocyanine green, indigo carmine, or methylene blue were frequently applied in peritoneal dialysis cases. Laparoscopic inspection was used in a limited number of cases when these methods failed.

The etiologies of PPC varied, with most cases being associated with peritoneal dialysis or hepatic hydrothorax. Our case, notably, was the only one reported following right hepatectomy (**Fig. 3**). In terms of preoperative diagnosis, peritoneal scintigraphy and contrast-enhanced CT peritoneography were commonly utilized in patients undergoing peritoneal dialysis. However, a substantial number of cases were diagnosed clinically, as was true in our patient (**Fig. 4A**).

Various intraoperative techniques have been employed to identify the diaphragmatic defect responsible for PPC. In peritoneal dialysis patients, indigo carmine or indocyanine green mixed with dialysate was frequently used. By contrast, pneumoperitoneum proved useful in several cases, including ours, by enabling direct visualization of the lesion. In rare cases, diagnostic laparoscopy from the peritoneal side was required when other methods failed (**Fig. 4B**).

To clarify the morphological spectrum of diaphragmatic lesions, we reclassified reported macroscopic findings into 6 categories, due to inconsistencies in terminology. The most common lesion type was bleb formation, followed by structural defects and microperforations (**Table 1**). Notably, no previous report described a pinhole defect overlying a hernia sac, suggesting a unique morphological presentation in our case (**Table 1**).

**Table 1:** Frequency and morphological classification of diaphragmatic lesions in PPC reported in the literature

Morphological category of diaphragmatic lesion	Representative terms in reports	Number of cases (n, %)	
Bleb-like lesion	Bleb	34 (43.0)	
Structural defect	Defect, tear, fissure, slit, perforation, fistula	23 (29.1)	
Microperforation	Pin-hole, small hole, small pore	15 (19.0)	
Fragile/thinned tissue	Thin membranous lesion, web, flaw	5 (6.3)	
Papillary lesion	Papillary lesion	1 (1.3)	
Herniation	Pin-hole defect in hernia sac	1 (1.3)	(Our case)

PPC, pleuroperitoneal communication

Surgical approaches for PPC varied widely. Repair techniques included direct suturing, stapler resection, reinforcement, and pleurodesis, applied either alone or in combination (**Table 2**). These findings underscore the lack of a standardized surgical strategy (**Table 2**).

**Table 2:** Treatment methods for pleuroperitoneal communication

Treatment method	Surgical	Non-surgical	Recurrence
Direct suture alone	7		1
+ Reinforcement	20		
+ Pleurodesis	19		1
+ Reinforcement + pleurodesis	2		
Stapler resection alone	10		1
+ Reinforcement	13		
+ Pleurodesis	1		
Reinforcement alone	5		
Pleurodesis alone	7	6	1
Discontinuation of PD	1	12	1
+ Pleurodesis		7	1
Thoracic drainage	1	2	
Others	3	2	
Not described	13		

PD, Peritoneal dialysis

In contrast to previously reported cases, PPC in our patient was associated with a pinhole defect in a diaphragmatic hernia. During the initial hepatectomy, no diaphragmatic abnormalities were observed intraoperatively, making a congenital anomaly unlikely. Although the patient later developed ovarian metastases and peritoneal carcinomatosis with significant ascitic fluid accumulation, pleural effusion did not occur at that time. Furthermore, pathological examination of the resected diaphragm showed no evidence of malignancy, ruling out direct tumor infiltration as a cause of diaphragmatic disruption. Ultimately, we concluded that PPC in this case resulted from a diaphragmatic hernia. As ascitic fluid accumulated, the hernia sac gradually expanded. The combined effects of increased intra-abdominal pressure due to ascitic fluid and negative intrathoracic pressure likely caused a pinhole defect to develop in the weakened, stretched diaphragm. This defect allowed the persistent migration of ascitic fluid into the thoracic cavity, resulting in pleural effusion.

Diaphragmatic hernia is a well-recognized complication following hepatectomy, often attributed to intraoperative diaphragmatic injury.^3)^ Although previous studies suggest that right-sided PPC may theoretically occur more frequently after hepatectomy due to incidental diaphragmatic damage,^1)^ no such case has been previously reported. Our literature review did not identify any similar cases, emphasizing the novelty of our report.

Regarding the surgical approach, a thoracoscopic approach was initially used to identify the hernia sac; however, the exact site of the fistula could not be confirmed. Therefore, a laparoscopic approach was added, which enabled precise identification of the defect under pneumoperitoneum and confirmation of the absence of herniated organs. These steps allowed safe resection of the hernia sac and repair of the diaphragm. Although the use of laparoscopy in PPC surgery is generally limited (**Fig. 4B**), this may be because most PPC cases occur on the right side, where the liver often obstructs visualization of the diaphragm. By contrast, after hepatectomy, laparoscopic visualization becomes easier, and the absence of adhesions beneath the diaphragm, as in this patient, allows safe access. When PPC is associated with a diaphragmatic hernia, as in this case, direct laparoscopic observation of the hernia sac before repair may contribute to procedural safety. This case suggests that a combined thoracoscopic and laparoscopic approach is an effective surgical option for PPC with diaphragmatic hernia following liver resection. Diaphragmatic repair can be performed using either a thoracoscopic or laparoscopic approach.^67)^ Both techniques are considered safe and are commonly performed in the routine clinical practice in Japan.^68)^

Recent studies on bronchopleural fistula repair have proposed stapling with reinforcement as a favorable technique, as it can withstand higher burst pressures.^69,70)^ Given the similarities in repair techniques, stapling with reinforcement may also be effective for diaphragmatic repair; however, further investigation is warranted to confirm its efficacy.

## CONCLUSIONS

PPC should be considered as a cause of right pleural effusion following right hepatectomy. When PPC is accompanied by a diaphragmatic hernia, intra-abdominal observation is desirable to assess the potential for organ herniation during diaphragmatic repair. Compared with PPC associated with peritoneal dialysis or hepatic hydrothorax, the diaphragm may be easier to evaluate after hepatic resection. Therefore, a combined thoracoscopic and laparoscopic approach appears to be a useful surgical option in such cases.

## DECLARATIONS

### Availability of data and materials

All related data are included within the article.
